# Contemporary Surgical Tricuspid Valve Repair

**DOI:** 10.3390/jcm15156022

**Published:** 2026-08-03

**Authors:** Vishnu Vasanthan, Mimi Deng, Elise Chan, Adeline Chan, Daniel Goubran, Vincent Chan

**Affiliations:** 1 Division of Cardiac Surgery, University of Ottawa Heart Institute, Ottawa, ON K1Y 4W7, Canada; 2 Division of Cardiac Surgery, University of Saskatchewan, Saskatoon, SK S7N 0W8, Canada; 3 Division of Cardiac Surgery, University of Toronto, Toronto, ON M5B 1W8, Canada; 4 Division of Cardiac Surgery, University of British Columbia, Vancouver, BC V6Z 1Y6, Canada

**Keywords:** heart failure, structural valve procedures, tricuspid regurgitation, tricuspid valve

## Abstract

**Background:** Tricuspid valve surgery is indicated for severe symptomatic tricuspid valve regurgitation (TR) when performed concomitant to another cardiac operation. While percutaneous therapies for TR continue to advance, anatomic constraints may necessitate consideration of surgery in some patients. **Methods**: Herein, we provide a contemporary review of surgical techniques used to treat TR, which can serve as a complement to existing and emerging tricuspid valve treatment therapies. **Results**: We performed a literature review on established surgical tricuspid repair techniques. **Conclusions**: Surgical repair of the tricuspid valve is safe and feasible. While annuloplasty remains the cornerstone operative strategy, leaflet and sub-valvular techniques serve as adjuncts to help successful repair. The therapeutic paradigm for TR continues to evolve.

## 1. Introduction

Tricuspid valve pathology is often less considered in adult patients for whom the dominant clinical focus centers on left-sided valve disease. Once considered the “forgotten valve,” intervention was frequently deferred until the onset of advanced right-sided heart failure. This therapeutic inertia stemmed from misconceptions that tricuspid regurgitation (TR) was either benign or would resolve following correction of left-sided lesions. Contemporary evidence, however, underscores that untreated TR is a major driver of morbidity, manifesting as progressive right ventricular (RV) dysfunction and systemic venous congestion [1,2].

Surgical repair has historically been the predominant approach to tricuspid valve intervention and favored over replacement due to avoidance of prosthesis-related complications [3,4]. Technical innovations, including modern annuloplasty rings, leaflet augmentation, and edge-to-edge repair, have expanded the operative repertoire. In parallel, the advent of minimally invasive and robotic platforms has extended candidacy, enabling reduced perioperative morbidity and broadening the scope of surgical management [5,6].

This review synthesizes contemporary surgical techniques for tricuspid valve repair, highlighting operative principles, patient selection, and outcomes. By integrating evidence from clinical trials, registries, and expert consensus, we aim to provide a comprehensive resource for surgeons and cardiologists navigating the evolving landscape of tricuspid valve intervention.

## 2. Methods

We conducted a qualitative literature review focusing on contemporary surgical techniques for tricuspid valve repair. The techniques described are common procedures employed during surgery and may have relevant considerations in the era of percutaneous therapies, especially those that may be performed after surgical tricuspid repair. We also provide a brief summary regarding the medical management of TR, along with catheter-based management options.

## 3. Results

### 3.1. Anatomy and Pathophysiology

The tricuspid valve is a complex structure comprising three leaflets (anterior, posterior, septal), a fibrous annulus, chordae tendineae, and papillary muscles. Its function is linked to RV geometry, function, and dynamics. The annulus is elliptical and dynamic, with significant dimensional changes during the cardiac cycle, with the anterior and posterior segments more prone to dilation [7].

The pathophysiology of TR can be broadly categorized into primary (organic) and secondary (functional) etiologies. Primary TR arises from intrinsic leaflet pathology such as rheumatic disease, endocarditis, carcinoid syndrome, or congenital malformations [8]. In these instances, leaflet repair or reconstruction may be required. Secondary TR, which constitutes most cases, results from annular dilation. As such, secondary TR may arise due to right ventricle dilation/dysfunction, pulmonary hypertension, left-sided valve disease, or atrial fibrillation [9].

Annular dilation, particularly along the anterior and posterior segments, leads to mal-coaptation of leaflets. Right ventricle enlargement exacerbates tethering, while atrial fibrillation contributes to annular dilatation through atrial remodeling. These mechanisms underscore the importance of tailoring surgical repair to the underlying pathology: annuloplasty for annular dilation, leaflet augmentation for tethering, and adjunctive techniques for complex anatomy [10].

Understanding the interplay between RV function, pulmonary pressures, and atrial dynamics is critical, as surgical outcomes are closely tied to the timing of intervention relative to RV remodeling [11].

### 3.2. Surgical Indications and Timing

Guidelines from the American Heart Association/American College of Cardiology (AHA/ACC) and the European Society of Cardiology (ESC) emphasize intervention for severe TR, particularly when concomitant left-sided valve surgery is planned [12,13]. Isolated tricuspid surgery is recommended for symptomatic severe TR refractory to medical therapy, provided RV function is preserved.

The timing of surgery is pivotal. Delayed intervention, after the onset of advanced RV dysfunction or end-organ damage, portends poor outcomes. Conversely, early repair has been shown to reduce progression of TR with favorable survival [14].

Key indications include severe TR with symptoms of right heart failure, progressive RV dilation or dysfunction, severe annular dilation (>40 mm or >21 mm/m^2^ indexed diameter) even with moderate TR, and concomitant left-sided valve surgery with moderate or greater TR [15]. Indeed, tricuspid valve repair may also be performed concomitant to mitral valve surgery at selected centers in patients with less than moderate TR, but in whom have annular dilation [3].

#### Incidence of Permanent Pacemaker Implantation

Thus, surgical decision-making requires integration of echocardiographic parameters, RV function, pulmonary pressures, and clinical trajectory [15,16,17,18,19]. As an adjunct to mitral valve surgery, concomitant tricuspid valve repair was associated with a lower incidence of reoperation for tricuspid regurgitation, progression of TR by two grades from baseline or the presence of severe TR, or death at 2 years [16]. However, the pacemaker rate in that trial was 14.1% at 2 years for patients that had concomitant tricuspid valve repair [16].

Retrospective data is also varied. In a study involving 1346 patients who underwent tricuspid valve surgery between 2011 and 2022 in Michigan, the rate of pacemaker implantation with any tricuspid operation was 11% [17]. In another large series involving 1058 patients who underwent tricuspid valve repair from 1997 to 2019, 10.3% received implantation of a permanent pacemaker [18]. In a large study involving data from Medicare fee-for-service beneficiaries aged 65–99 years with TR in the United States, patients who underwent surgery had a permanent pacemaker implantation rate of 12.7% [19].

### 3.3. Preoperative Assessment and Imaging

Comprehensive preoperative imaging is essential for surgical planning. Transthoracic echocardiography (TTE) provides initial assessment of TR severity, RV size and function, and pulmonary pressures [20]. Transesophageal echocardiography (TEE), including 3D TEE, offers superior leaflet visualization, quantification of regurgitant orifice, and intraoperative guidance [20,21]. Indeed, TEE is critical in evaluating the feasibility of percutaneous repair or replacement since leaflet visualization with or without the aid of intracardiac echocardiography is paramount [21]. Cardiac CT can delineate annular geometry, calcification, and spatial relationships relevant to ring sizing and coronary artery proximity [22]. Cardiac MRI is the gold standard for RV volume assessment and may be particularly useful when echocardiographic windows are limited. Strain imaging and speckle tracking may also help evaluate/detect RV dysfunction and help time intervention before irreversible remodeling. Integration of multimodality imaging with clinical assessment is critical in determining the indication and timing of intervention. Although biochemical tests, such as those that evaluate liver function, are germane to tricuspid regurgitation evaluation, biomarkers of heart failure are less evaluated in the absence of concomitant left-sided heart disease [22,23,24,25].

### 3.4. Surgical Techniques

#### 3.4.1. Overview

Surgical tricuspid valve repair can be performed via sternotomy or mini-thoracotomy/port access. This involves cardiopulmonary bypass, but can be performed without or without cardiac anoxia.

#### 3.4.2. Annuloplasty

**Figure 1:**
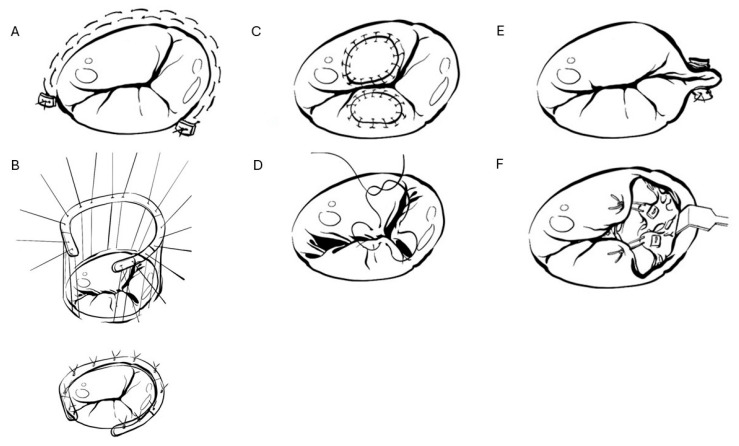
**Surgical tricuspid valve repair techniques.** These include (**A**) suture annuloplasty, (**B**) ring annuloplasty, (**C**) patch augmentation, (**D**) edge-to-edge repair, (**E**) bicuspidization, and (**F**) chordal replacement.

Annuloplasty is the cornerstone of tricuspid valve repair. The principle is reduction of annular dimensions to restore leaflet coaptation. Suture annuloplasty techniques, such as those described by Kay and De Vega, were first described several decades ago (Figure 1A). While technically simple, these methods suffer from high TR recurrence rates [26]. Suture annuloplasty, however, can be performed efficiently and has the advantage of less prosthetic material, which may be advantageous in the setting of active infection. Ring annuloplasty is the surgical gold standard. Rigid and semi-rigid rings remodel the annulus into a physiologic three-dimensional shape, allowing for leaflet coaptation (Figure 1B). Contemporary bands are more flexible and may limit disruptions of the atrioventricular node since they accommodate for the non-planar shape of the tricuspid annulus [23]. Technical pearls include careful suture placement in the annulus while avoiding the conducting tissue located within the triangle of Koch [27]. Comparative studies consistently demonstrate superior outcomes with ring annuloplasty, including lower TR recurrence rates. Although the need for permanent pacemaker implantation may approach 14% in some patients, others have reported a lower rate in select patients [28].

#### 3.4.3. Leaflet Augmentation

Leaflet augmentation may be used to address tethering due to RV dilation. Autologous pericardial patches are used to enlarge leaflet surface area, improving free-margin coaptation (Figure 1C). This technique may be applied in patients with functional TR with severe tethering. Leaflet augmentation may also be useful to replace portions of the leaflet body that may be calcified, sclerotic as in rheumatic disease or congenital malformations, or involve vegetation or infection. Outcomes are challenging since the underlying pathophysiology is different than for patients with simply functional TR due to annular dilation [29]. Long-term outcomes therefore depend on the underlying disease process and the incorporation of the pericardial patch [29].

#### 3.4.4. Edge-to-Edge Repair

Adapted from the Alfieri stitch used in mitral surgery, edge-to-edge repair involves suturing leaflet edges to create a triple-orifice, or cloverleaf, valve. This technique is useful in central regurgitant jets or complex anatomy involving a combination of either prolapse or restriction. (Figure 1D) [30].

#### 3.4.5. Adjunctive Techniques

Adjunctive methods include bicuspidization, whereby sutures are placed to exclude the posterior tricuspid valve leaflet (Figure 1E), chordal shortening, papillary muscle relocation, neochordal replacement (Figure 1D), and right atrial reduction. These are reserved for complex cases with severe tethering or distortion. While technically demanding, they can enhance durability when combined with annuloplasty [31,32]. It is important to note that the chordae that supply the anterior and posterior leaflets come from the anterior papillary muscle. Therefore, care must be given to chordal supports in the region of the neo-commissure with bicuspidization [31,32].

#### 3.4.6. Minimally Invasive and Robotic Approaches

Minimally invasive approaches via right mini-thoracotomy or port-access techniques have gained traction, offering reduced morbidity and faster recovery. Robotic annuloplasty provides enhanced visualization and precision, though adoption remains limited to specialized centers. Early outcomes are promising, with comparable durability to sternotomy approaches [33,34,35].

### 3.5. Intraoperative Considerations

Intraoperative TEE is indispensable for confirming mechanisms, guiding repair, and assessing immediate results. Key steps include reassessment of leaflet mobility after cardioplegia, trial annuloplasty sizing with saline testing, and iterative adjustments to achieve optimal coaptation. Avoiding excessive septal annular suturing (towards the antero-septal commissure) reduces conduction injury risk. When performing leaflet augmentation or chordal work, ensure secure patch fixation and tension-free neochordal placement. Hemodynamic optimization and careful deairing are essential before weaning from cardiopulmonary bypass.

## 4. Isolated Tricuspid Regurgitation and Emerging Technology

Transcatheter therapies are evolving to treat TR. Evidence from large registries suggests that patients with advanced right heart symptoms may not benefit from surgery. Indeed, a TRISCORE of ≥6, which takes into consideration age, symptom status, right heart failure symptoms, liver function, kidney function and degree of diuretic, was associated with poor outcomes [11]. Contemporary retrospective data suggests a crude perioperative surgical mortality of approximately 7% with a 20% pacemaker implantation rate [36]. Registry data describing transcatheter edge-to-edge repair may be associated with 2–3% mortality [37,38].

Transcatheter tricuspid valve repair via percutaneous edge-to-edge repair (T-TEER) is effective. Data from the TRILUMINATE pivotal trial showed that T-TEER and optimized medical therapy was better than medical therapy alone in reducing TR and improving the Kansas City Cardiomyopathy Questionnaire [39,40]. The Tri-fr investigators found similar results 1 year after T-TEER [41]. Tricuspid edge-to-edge repair is performed using either the TriClip (Abbott Cardiovascular, MN, USA) [39,40] or PASCAL (Ewards Lifesciences, CA, USA) device [42]. However, there may be limitations regarding its use in select patients with a large leaflet gap. In such patients, transcatheter tricuspid valve replacement has shown early promise. The EVOQUE valve has shown promise with a 7% 1-year mortality involving 27 patients who underwent the procedure for compassionate use [43]. Additionally, evolving technology such as the TricValve, which involves placement of a bovine valve in the superior and inferior vena cavae, has shown promise.

## 5. Comment

Tricuspid valve regurgitation may occur due to organic or functional disease [1,2,3,4,5,6,7,8,9,10]. Although most often managed medically, severe TR is increasingly treated with catheter-based approaches. Although percutaneous repair is the most likely treatment modality, surgery may still be required in selected patients. In addition, surgical techniques used to treat TR are varied, and these techniques may serve to inform outcomes following analogous catheter-based procedures. Ultimately, long-term outcomes will depend on patient anatomy.

The advent of the TRI-SCORE highlights this principle. Indeed, compared to conservative management, patients who underwent tricuspid intervention with a lower comorbid burden (TRI-SCORE ≤ 3) had better survival. Patients with a high TRI-SCORE ≥ 6 did poorly regardless of treatment or treatment type [11].

For specific surgical repair techniques, it is challenging to ascertain specific outcomes. The surgical literature often includes patients with mixed-etiology TR, and therefore results are often described based on technique alone.

Nevertheless, in a cohort of 624 functional TR patients undergoing concomitant mitral valve surgery, TR > 2+ was associated with worse survival over the long term [3]. For patients with endocarditis, a meta-analysis of tricuspid surgery patients showed that tricuspid valve repair performed with suture or ring annuloplasty, bicuspidization, or patch augmentation was associated with a lower rate of recurrent endocarditis, but higher risk of recurrent moderate–severe TR [44].

Chordal tricuspid valve repair was associated with a 3-year culminative risk of tricuspid valve reintervention of 7.3%, although 40% of the cohort comprised patients with a congenitally malformed valve [45].

Tricuspid edge-to-edge repair is reported to have a culminative incidence function of TR ≥ 2+ at 16 years of 24%, when considering death as a competing risk. This cohort of 145 patients included a mixed cohort of patients with TR due to prolapse, annular dilation, and tethering [30].

Overall, what predicts recurrent TR over time depends on preoperative and late postoperative patient factors [3,4,5,6]. Right ventricle dysfunction, severe pulmonary hypertension, and atrial fibrillation tend to be associated with TR after repair [3,4,5,6].

### Future Directions

The future of tricuspid valve repair lies in integration with transcatheter therapies. Transcatheter edge-to-edge repair has emerged as a viable treatment option for select patients with severe TR [37,38,39,40].

Optimal management of TR, however, extends beyond the specific technology and may benefit from a multidisciplinary heart team approach involving cardiac surgeons, interventional cardiologists, imaging specialists, heart failure clinicians, and anesthesiologists. Multidisciplinary case review also ensures appropriate selection between medical, surgical and transcatheter options, timing of intervention, and perioperative optimization. Prehabilitation, arrhythmia management (including consideration of concomitant atrial fibrillation ablation), and postoperative surveillance are integral to improving outcomes.

Refinement of patient selection algorithms, incorporating advanced imaging and biomarkers, will optimize timing and technique. Patient-reported outcomes may also be useful in guiding therapy. Multicenter registries and randomized trials are needed to define best practices and long-term outcomes. At present, there are no longitudinal datasets that directly compare specific surgical repair strategies with contemporary transcatheter valve reconstruction techniques. This gap underscores the critical need for robust, long-term registry infrastructures capable of evaluating comparative effectiveness across evolving therapeutic modalities.

## 6. Conclusions

Surgical tricuspid valve repair remains a durable and common option for treating primary and secondary TR. Annuloplasty is the cornerstone, but adjunctive leaflet techniques expand the surgeon’s ability to address complex pathology. As transcatheter therapies mature, a collaborative, heart team approach will be essential to match each patient with the most appropriate, durable therapy. Continued innovation in devices, imaging, and trial design will refine indications and improve long-term outcomes. This review of surgical techniques may therefore be especially relevant given the potential for the catheter-based treatment of a patient with previous surgical tricuspid valve repair. Nevertheless, early intervention—guided by multimodality imaging and risk stratification—optimizes outcomes by preventing irreversible RV remodeling.

## 7. Key Takeaways

- Surgical repair remains a viable strategy to treat tricuspid regurgitation, with annuloplasty as the cornerstone supplemented by adjunctive leaflet/sub-valvular techniques.
- Advances in minimally invasive and robotic approaches have broadened surgical options.
- Future directions emphasize integration with transcatheter therapies and refined patient selection, underscoring an evolving paradigm in tricuspid valve intervention.
- A heart team model may benefit patients by including a multidisciplinary team that involves imaging specialists, anesthesiologists, heart failure cardiologists and rhythm specialists.

## Data Availability

No new data were created or analyzed in this study. Data sharing is not applicable to this article.
